# Asian cultural values and help-seeking: a cross-sectional study on compulsive sexual behavior

**DOI:** 10.3389/fpsyt.2025.1633160

**Published:** 2025-10-07

**Authors:** Hoang-Long Pham, Vu Hoang Anh Nguyen, Cong Minh Le, Thanh-Ha Truong-Thai, Quynh-Anh Vu-Thi, Xuan-Phat Phan, Hoa-Nhu Nguyen-Vo, Thuy Doan Hua, Bao-Tran Nguyen-Duong, Cyrus Su Hui Ho, Vinh-Long Tran-Chi

**Affiliations:** ^1^ Faculty of Psychology, Ho Chi Minh City University of Education, Ho Chi Minh City, Vietnam; ^2^ Department of Psychosomatics, Thu Duc City Hospital, Ho Chi Minh City, Vietnam; ^3^ University of Social Sciences and Humanities, Ho Chi Minh City, Vietnam; ^4^ Vietnam National University, Ho Chi Minh City, Vietnam; ^5^ Department of Psychological Medicine, National University of Singapore, Singapore, Singapore; ^6^ Department of Psychological Medicine, National University Hospital, Singapore, Singapore

**Keywords:** Asian values, compulsive sexual behavior, help-seeking attitudes, psychological help-seeking, CSB

## Abstract

**Background:**

Sexuality is a complex construct influenced by various social and cultural factors, and while sexual behavior is generally considered adaptive for physical and mental well-being, it can become uncontrollable and harmful, leading to Compulsive Sexual Behavior (CSB). Limited research has explored how Asian cultural values may mediate the psychological consequences of CSB and influence attitudes toward seeking professional psychological help. This study examines the relationships among CSB, Asian cultural values, and attitudes toward seeking professional psychological help (ATSPPH) in a Vietnamese context.

**Methods:**

Three scales were employed to gather demographic variables and self-reported data, including the Asian Values Scale – Revised, the Individual-Based Compulsive Sexual Behavior Scale, and Attitudes Toward Seeking Professional Psychological Help. The present study was evaluated utilizing the partial least squares-structural equation modeling (PLS-SEM) methodology.

**Results:**

The findings confirm significant relationships, with a lack of control and negative affect in CSB being negatively associated with freedom and flexibility in Asian values. Conversely, the negative consequences of CSB showed a positive relationship with these values. Crucially, both traditional and freedom-flexibility values were found to negatively predict openness to seeking professional help. The study also revealed that Asian cultural values play a significant mediating role between CSB and ATSPPH. Furthermore, behavioral factors such as the frequency of pornography use, sexual frequency, and having a stable sexual partner moderated these relationships.

**Discussion:**

These results highlight the complex interplay of cultural beliefs and individual behavior, underscoring the need for culturally sensitive interventions for those struggling with CSB in Asian culture.

## 1 Introduction

Sexuality is a complex construct encompassing individual, familial, societal, and cultural characteristics, including sexual pleasure, gender identity, attitudes, and behaviors, all shaped by both proximal and distal social influences such as family, peers, and broader cultural contexts (1). In general, sexual behavior is regarded as an adaptive form of behavior that serves multiple biological and social functions. These include satisfying physiological needs, strengthening interpersonal bonds, and regulating emotional states such as pleasure, satisfaction, stress relief, and feelings of loneliness (2, 3). The benefits of sexual behavior extend beyond procreation to include positive effects on emotional stability, stress resilience, and mental health (4). However, in certain cases, these adaptive behaviors may become repetitive, difficult to control, and potentially harmful, even when individuals remain aware of their actions but struggle to stop them (5).

Compulsive sexual behavior (CSB) refers to persistent, uncontrolled sexual behaviors and is also known as sex addiction, hypersexuality, or compulsive sexual behavior disorder (6). CSB is defined by persistent sexual fantasies, urges, or behaviors that cause significant psychological distress and interfere with personal and social functioning (7, 8). According to ICD-11, CSB is classified as an impulse control disorder, characterized by a continued inability to control intense sexual impulses lasting at least six months and leading to substantial impairment in key areas of daily life (9, 10). Notably, the motivational mechanisms underlying CSB often shift as the disorder develops: the initial pursuit of sexual pleasure may gradually take on the function of coping with stress and negative emotions, reflecting a transition from liking to wanting similar to mechanisms identified in other addictive disorders (3, 11). Many studies have found that individuals with CSB are more likely to experience mood and anxiety disorders, impulse control difficulties, and substance abuse or addiction, all of which can result in serious negative consequences (12). Although professional support is often needed, many individuals remain reluctant to seek psychological help due to feelings of shame, guilt, or emotional distress (13, 14).

Attitudes toward seeking professional psychological help (ATSPPH) is a multidimensional concept encompassing an individual’s willingness, expectations, and perceptions about seeking support from mental health professionals (15, 16). These attitudes, which comprise varying degrees of openness to therapy, perceived stigma, and a preference for self-reliance, are often exacerbated by stigma, a lack of awareness about available services, and cultural norms that discourage open discussion of mental health issues (17). Consequently, individuals may internalize their struggles, leading to prolonged suffering and increased symptom severity (17). This hesitation delays treatment and worsens CSB, as individuals keep engaging in harmful sexual behaviors to cope, leading to more distress and anxiety (18). As a result, despite recognizing the loss of control over their sexual behavior, individuals often face multiple barriers that hinder help-seeking efforts (18). Kraus, Martino and Potenza (8) found that one in seven men expressed a wish to receive help in managing their pornography use, which is a significant predictor of CSB. However, only a small proportion acted on this intention, with just 6.4% seeking help (8). Among those who did seek support, nearly half continued to report a need for ongoing professional assistance (8). These findings indicate that even after treatment, many individuals feel that continuous professional guidance is necessary to fully address the challenges related to CSB.

Importantly, the compulsive aspect of CSB differs from typical compulsive behaviors in that individuals often seek novel and risky sexual experiences rather than rigidly repeating the same acts (19). Research has also highlighted the wide range of behavioral expressions among individuals with CSB, including excessive masturbation, pornography use, phone sex, cybersex, visiting strip clubs, paying for sexual services, or engaging in persistent sexual fantasies (20, 21). Moreover, problematic pornography users tend to hold more negative attitudes about their behavior compared to those who view pornography purely for recreation (22, 23).

Although the behavioral and motivational features of CSB have been explored in depth, debate remains regarding the role of pornography use in reinforcing compulsivity and behavioral dysregulation (24). Some perspectives argue that pornography is inherently an addictive medium that inevitably contributes to increased compulsive behavior and related disorders (25). Furthermore, several studies have reported consensus that pornography use can pose serious problems, with some individuals experiencing severe consequences that significantly diminish their quality of life (26). It is also important to note that the diagnosis of sexual disorders in general, and CSB in particular, should not rely solely on behavioral symptoms but must be considered within the broader socio-cultural context (9).

In addition to individual factors and behavioral characteristics, the cultural context is widely regarded as a key element shaping sexual attitudes and behaviors (27). Research into sexuality is often hindered by the profound cultural sensitivity and social taboos surrounding sexuality that persist in many Asian contexts (28). Asian culture is strongly influenced by Confucianism, which emphasizes social harmony, hierarchical roles, and collective responsibility (29) is frequently conceptualized along the dimensions of individualism and collectivism within cross-cultural psychology (30, 31). These constructs represent fundamental differences in how individuals relate to groups and prioritize personal versus collective goals, providing a robust framework for understanding cultural variations in attitudes, cognition, and behavior (32). These values, rooted in collectivism, tend to discourage premarital sexual activities because they are seen as violating established social norms (33). In contrast, individualism as a cultural orientation that prioritizes individual needs and desires over collective interests is frequently linked to a loosening of social norms related to marriage and sexuality (34). At its core, marriage is traditionally viewed as a socially sanctioned form of legal sexual relationship, whereas cultural individualism highlights personal autonomy, placing less weight on community norms and collective recognition, which may increase acceptance of extramarital sexual behaviors (35). Several studies have shown that higher levels of individualism are associated with more permissive attitudes toward pornography consumption, premarital sex, and general sexual permissiveness (36, 37).

In Asian contexts where traditional values are dominant, individuals with CSB characterized by a loss of control over sexual behaviors that become potentially harmful often endure internalized shame and psychological conflict (28). Such internal conflict can influence how individuals negotiate their Asian values, shaping whether they continue to follow established moral frameworks or adopt more flexible positions aligned with cultural norms (38). Asian cultural values significantly shape beliefs about seeking professional psychological help, including attitudes toward mental health, individual responsibility, and stigma related to therapy (39–42). Those who strictly adhere to traditional values may perceive psychological distress as a private burden that should be resolved independently rather than through professional intervention (43–46). This tendency partly reflects the view that acknowledging mental health concerns is sometimes considered a collective failure of the family or extended kinship networks in Asian societies (47). Therefore, collectivism or familialism key features of Asian values may predict a reluctance to pursue professional treatment for mental health conditions (48). Conversely, individuals whose values are more flexible may demonstrate a greater willingness to seek help, as they are less constrained by cultural stigma associated with mental health care (49).

In Vietnamese culture, discussions surrounding sex and sexual behavior remain profoundly sensitive, heavily influenced by Confucian values. This ideological system upholds strict moral standards for sexual conduct, particularly emphasizing premarital chastity and the purity of women (50). Consequently, topics related to sexuality are often addressed only indirectly through literary works or cultural documents (50). Within this context, sexuality is perceived not merely as a pure instinct but as a socio-cultural construct, subject to control and influence by prevailing social norms (51). From this perspective, dominant cultural discourses primarily shape sexual activity around duty and morality, rather than intimacy or personal well-being (52). Specifically, sexual acts are often viewed as a marital obligation or a means to ensure physical harmony before marriage, rarely as a legitimate expression of love or pleasure. Consequently, terms such as “sex addiction,” “sexual deviance,” “promiscuous lifestyle,” or “uncontrollable sexual desire” are seldom openly discussed. Notably, the term CSB is yet to be standardized and officially recognized within the clinical and academic communities in Vietnam.

Sexual perceptions in Vietnam have undergone notable shifts, particularly among youth, with a higher prevalence of sexual relations, including premarital sex (50). Data shows the average age of first sexual intercourse has decreased to 18.7 years (53), with a study on first-year college students finding that 16.1% had engaged in premarital sex. A concerning 15% of students reported having had sexual intercourse before the age of 18, which is considered early sexual activity and is linked to various potential consequences (54). University students, especially those living independently and having diverse social relationships, are identified as a high-risk group for early and premarital sexual activities (54). These changes in behavior are accompanied by a significant gender disparity in the acceptance of extramarital relations, with men often receiving greater leniency based on traditional beliefs about their sexual needs (50, 55–60). These evolving behaviors and perceptions have a direct impact on public health, as Vietnam records over 200,000 cases of sexual health issues annually, with nearly half of them occurring in individuals aged 16 to 29 (61). Furthermore, the link between mental and sexual health is evident, with studies reporting a high prevalence of sexual dysfunction among patients with depression (53.8% in men and 78.2% in women) (62).

Despite previous studies examining the relationship between cultural values and ATSPPH, a notable gap exists in directly addressing how Asian cultural norms influence CSB and ATSPPH specifically in the Vietnamese context. While CSB research has been conducted across various regions, evidence remains limited on how these behaviors are shaped by Vietnam’s unique socio-cultural environment and its evolving sexual landscape. This dearth of clear terminology and public understanding concerning sexual behavior issues may contribute to limited awareness and reinforce existing stigma. Furthermore, when combined with strong collectivist norms that emphasize family reputation and social harmony, this conceptual and societal gap may significantly discourage individuals from acknowledging problematic sexual behaviors or seeking appropriate professional psychological support.

To address these gaps, this study purposes to investigate the relationships among Asian cultural values, CSB, and ATSPPH within the Vietnamese cultural framework. Specifically, the study examines how key dimensions of CSB are associated with Asian values and ATSPPH, while accounting for the potential moderating effects of sexual frequency, stable partnerships, and pornography consumption. Furthermore, it explores whether Asian cultural values mediated the relationship between CSB and ATSPPH. The findings are expected to provide insights for developing culturally sensitive interventions and mental health services that are responsive to the specific needs and cultural contexts of Vietnamese young adults.

Based on these purposes, the study focuses on the following core research questions:

1. How are the key dimensions of CSB related to Asian cultural values among undergraduate students?
2. How do Asian cultural values relate to ATSPPH, and do they mediate the relationship between CSB and ATSPPH?
3. To what extent do factors such as pornography consumption, sexual frequency, and having a stable sexual partner moderate these relationships?

Accordingly, the study proposes the following hypotheses:

H1: Each dimension of CSB is significantly associated with aspects of Asian cultural values, such as freedom, flexibility, and adherence to traditional norms.
H2: Each aspect of Asian cultural values is significantly related to ATSPPH.
H3: Each dimension of CSB is directly associated with ATSPPH.
H4: Frequency of pornography use in the past seven days moderates the relationship between negative affect (a dimension of CSB) and perceived need for professional help.
H5: The time spent on pornography moderates the relationship between adherence to traditional values and perceived need for psychological support.
H6: Sexual frequency moderates the relationship between negative affect (a dimension of CSB) and adherence to traditional norms.
H7: Having a stable sexual partner moderates the relationship between the unwanted consequences (a dimension of CSB) and freedom and flexibility values.
H8: The dimensions of CSB indirectly affect perceived ATSPPH through their influence on Asian cultural values.

## 2 Materials and methods

### 2.1 Data collection

This study was conducted as a cross-sectional descriptive analysis, with invitations extended to all university students across Vietnam. The selection criteria encompassed students from various academic disciplines who voluntarily participated in the survey. Among the 591 respondents, 232 were excluded due to incomplete responses. Consequently, the final sample size for the study comprised 359 participants.

Data were collected from November 23rd to December 15th, 2024, using an online survey administered via Google Forms, which required approximately 15–20 minutes to complete. A multi-pronged recruitment strategy was implemented to reach participants. This strategy included: (1) in-person visits to university campuses in Ho Chi Minh City, where research team members approached students and provided a direct link or QR code to the survey; (2) online distribution through social media student groups; and (3) collaboration with faculty members at universities in Northern Vietnam to disseminate the survey link to their students. Informed consent was electronically obtained from all participants prior to their engagement with the survey.

Among the participants (over 18 years), 135 (37.6%) were males and 224 (62.4%) were females. Participants’ academic levels were freshman (n = 123; 34.3%), sophomore (n = 45; 12.5%), junior (n = 118; 32.9%), and senior or above (n = 73; 20.3%). The participants’ sexual orientations were Heterosexual (n = 212; 59.1%), LGBTQ+ (n = 147; 40.9%), as presented in **Table 1**.

**Table 1:** Demographic characteristics.

Variable		Total (n= 359) Frequency (%)	ICSB (n=359) Mean ± SD	AV (n=359) Mean ± SD	ATSPPH (n=359) Mean ± SD
Gender	Male	135 (37.6)	3.06 ± 1.2	2.45 ± 0.25	2.99 ± 0.49
	Female	224 (62.4)	2.31 ± 1.01	2.31 ± 0.28	3.20 ± 0.43
Sexual Orientation	Heterosexual	212 (59.1)	2.53 ± 1.08	2.39 ± 0.25	3.12 ± 0.48
	LGBTQ+	147 (40.9)	2.67 ± 1.23	2.33 ± 0.29	3.13 ± 0.44
Academic Levels	Freshman	123 (34.3)	2.43 ± 1.17	2.39 ± 0.26	3.04 ± 0.46
	Sophomore	45 (12.5)	2.90 ± 1.33	2.38 ± 0.25	3.19 ± 0.45
	Junior	118 (32.9)	2.64 ± 1.13	2.34 ± 0.27	3.14 ± 0.46
	Senior or above	73 (20.3)	2.61 ± 0.95	2.34 ± 0.31	3.18 ± 0.47
Academic field	Education	59 (16.4)	2.43 ± 1.20	2.39 ± 0.25	3.11 ± 0.40
	Humanities	161 (44.8)	2.63 ± 1.13	2.31 ± 0.28	3.20 ± 0.50
	Business	37 (10.3)	2.71 ± 1.15	2.36 ± 0.31	2.98 ± 0.42
	Natural sciences	25 (7.0)	2.65 ± 1.19	2.44 ± 0.25	3.02 ± 0.42
	Engineering & Technology	61 (17.0)	2.57 ± 1.12	2.43 ± 0.25	3.06 ± 0.44
	Other	16 (4.5)	2.51 ± 1.07	2.48 ± 0.21	3.05 ± 0.49
Area	Urban area	285 (79.4)	2.59 ± 1.12	2.35 ± 0.29	3.12 ± 0.48
	Rural area	74 (20.6)	2.60 ± 1.23	2.41 ± 0.21	3.10 ± 0.40
Religion	No religion/belief	158 (44.0)	2.45 ± 1.06	2.37 ± 0.28	3.07 ± 0.52
	Practice of religion/belief	67 (18.7)	2.86 ± 1.26	2.42 ± 0.26	3.16 ± 0.42
	No practice but belief	134 (37.3)	2.63 ± 1.17	2.33 ± 0.27	3.17 ± 0.43
Stable Partner	Not sexually active	258 (71.9)	2.51 ± 1.10	2.37 ± 0.27	3.12 ± 0.47
	Have a stable sexual partner	59 (16.4)	2.91 ± 1.18	2.35 ± 0.25	3.10 ± 0.49
	Do not have a stable sexual partner	42 (11.7)	2.61 ± 1.28	2.37 ± 0.30	3.13 ± 0.39
Sexual Frequency	Never had sexual activity	261 (72.7)	2.48 ± 1.12	2.37 ± 0.27	3.13 ± 0.47
	Less than 10 times a year	32 (8.9)	2.91 ± 1.01	2.35 ± 0.31	3.11 ± 0.43
	1–3 times per month	35 (9.7)	2.81 ± 1.02	2.31 ± 0.25	3.15 ± 0.38
	Once a week or more	31 (8.6)	2.94 ± 1.45	2.39 ± 0.25	3.05 ± 0.54
Pornography Time 7 days	None	210 (58.5)	2.30 ± 0.99	2.37 ± 0.27	3.14 ± 0.43
	59 minutes or less	114 (31.8)	2.79 ± 1.16	2.36 ± 0.27	3.19 ± 0.43
	60–119 minutes	19 (5.3)	3.39 ± 0.83	2.26 ± 0.32	2.77 ± 0.54
	120 minutes and more	16 (4.5)	4.08 ± 1.32	2.52 ± 0.21	2.80 ± 0.72
Pornography Frequency 7 days	None	215(59.9)	2.31 ± 0.99	2.37 ± 0.28	3.15 ± 0.43
	1–3 times	107 (29.8)	2.78 ± 1.12	2.35 ± 0.29	3.12 ± 0.49
	4–6 times	20 (5.6)	3.51 ± 1.02	2.39 ± 0.20	2.95 ± 0.50
	More than 6 times	17 (4.7)	3.91 ± 1.51	2.41 ± 0.24	2.92 ± 0.64

ICSB, Individual Compulsive Sexual Behavior; AV, Asian Values; ATSPPH, Attitude Toward Seeking Psychology Profession Help.

Informed consent was obtained from all participants before their involvement in the survey. Participation was entirely voluntary, with no remuneration provided, and respondents retained the right to withdraw from the study at any point during the data collection process. Participants with any questions were encouraged to contact the research team via email. They were fully informed about the study’s objectives and were asked to provide sociodemographic information, including gender, sexual orientation, academic level, department, area, religion, household income, monthly expenditure, part-time job, counseling experience and some questions about their sexual activities.

The Asian Values Scale – Revised (25 items) (63) and the Individual-based Compulsive Sexual Behavior Scale (64) were translated into Vietnamese and English through a forward and backward translation process conducted by four professional translators. The team included four native Vietnamese speakers fluent in English. The backward translation team currently resides in foreign countries and has never been exposed to the original scale. During this process, the authors encountered particular challenges in finding Vietnamese equivalents for key constructs, for instance, rendering “compulsive sexual behavior” accurately as “*hành vi tình dục cưỡng chế*” without implying moral judgment, and capturing the nuance of “emotional restraint” in AVS-R items so that social appropriateness was preserved without suggesting pathological suppression of feelings. After comparing the back-translations to the originals, the research team held consensus meetings to resolve discrepancies, discussing cultural connotations of critical phrases and prioritizing conceptual equivalence over literal word-for-word matching. Finally, the entire team evaluated all translations for content accuracy and consistency, ensuring that each item would elicit the same psychological construct in Vietnamese respondents as intended in the original scales.

### 2.2 Measurement of variables

#### 2.2.1 The Individual-based Compulsive Sexual Behavior

Compulsive sexual behavior was assessed using the Hebrew version of the Individual-based Compulsive Sexual Behavior Scale (I-CSB (64);). The I-CSB was developed to capture distinct components of compulsive sexual behavior, including sexual fantasies, obsessive sexual thoughts, and excessive time spent consuming pornography. This self-report instrument consists of 24 items, measuring four latent dimensions: unwanted consequences (e.g., “I feel that my sexual fantasies hurt those around me”), lack of control (e.g., “I waste lots of time with my sexual fantasies”), negative affect (e.g., “I feel bad when I don’t manage to control my sexual urges”), and affect regulation (e.g., “I turn to sexual fantasies as a way to cope with my problems”). Participants rated each item on a 7-point Likert scale, ranging from 1 (not at all) to 7 (very much). Cronbach’s α values were.87 for unwanted consequences,.87 for lack of control,.80 for negative affect, and.84 for affect regulation. A total CSB score was also computed by averaging the 24 I-CSB items (Cronbach’s α = .94). To support the construct validity of the scale, model refinement was conducted by adding theoretically justified error covariances for residuals exceeding |0.30|, consistent with recommendations by Brown (65) and Byrne (66), resulting in an acceptable model fit (χ²/df = 3.19, RMSEA = 0.078, SRMR = 0.054, CFI = 0.933, TLI = 0.888). This analytical procedure showed that the ICSB was psychologically valid and appropriate for measuring compulsive sexual behavior in this group of people. The high internal reliability of the subscales made it possible to use them solely in further analyses.

#### 2.2.2 The Asian Values-Revised

The Asian Values Scale – Revised (AVS-R) is a 25-item self-report instrument designed to assess endorsement of traditional Asian cultural values (67), adapted from the original 36-item version (Kim et al., 1999). Items are rated on a 4-point Likert scale ranging from 1 (strongly disagree) to 4 (strongly agree), with sample items such as “One should not deviate from familial and social norms.” In the present study, the AVS-R was divided into two theoretically grounded subscales – Traditional Norms (13 items) and Freedom & Flexibility (12 items) – based on Hofstede (30), Hofstede (68) collectivism–individualism framework. Internal consistency reliability was acceptable, with Cronbach’s α = .79 for Traditional Norms, α = .80 for Freedom & Flexibility, and α = .655 for the full scale. Confirmatory factor analysis (CFA) conducted on a sample of 359 Vietnamese university students yielded the following model fit indices: χ²(274) = 774.891, χ²/df = 2.828, RMSEA = 0.071, SRMR = 0.081, CFI = 0.741, and TLI = 0.717, indicating a modest yet acceptable fit of the two-factor model in this cultural context (65, 66).

#### 2.2.3 Attitudes toward Seeking Professional Psychological Help

The ATSPPH-SF is a brief, 10-item survey designed to assess (1) individuals’ openness to seeking support and (2) their perceived need to consult mental health professionals when experiencing psychological difficulties (69). Each item was responded to on a 5-point Likert scale ranging from 1 to 5 (strongly disagree to strongly agree) (e.g., “I might want to have psychological counseling in the future”). The ATSPPH-SF in this study was measured using the Vietnamese translation adapted Tran-Chi, Ly (70) to examine young adults’ attitudes toward seeking professional psychological help.

In this study, Cronbach’s alpha for the overall scale was lower than 0.6 (0.491), and the alpha coefficients for the two dimensions were 0.51, and 0.79. Cronbach’s alpha should exceed 0.70 to confirm scale reliability; nevertheless, values below 0.70 remain acceptable (71, 72). An item-total correlation analysis was conducted to examine the psychometric quality in deeper terms. Items exhibiting poor corrected correlations (< 0.30) were designated for future enhancement; however, they were retained for this analysis due to the conceptual significance of the whole scale. The decision to keep both subscales was grounded in their theoretical significance and prior validation.

### 2.3 Data analysis

This study involved data collection, which was then encoded, cleaned, and adjusted for errors utilizing Excel software. A data analysis was conducted utilizing two software applications: SPSS version 26 and PLS-SEM version 4.0. In SPSS 26, we evaluated normality and utilized T-tests and ANOVA to analyze the differences among the variables in this research. The relationship between constructs examined with PLS-SEM involves assessing both the measurement model and the structural model.

## 3 Results

### 3.1 Demographic characteristics

The study included 359 participants, with 37.6% identifying as male and 62.4% as female. Most participants (59.1%) identified as heterosexual, while 40.9% identified as LGBTQ+. A majority were juniors (32.9%) or freshmen (34.3%), and most resided in urban areas (79.4%). In terms of relationship stability, 71.9% of participants were not sexually active, 16.4% had a stable sexual partner, and 11.7% did not have a stable partner. Sexual frequency data indicated that 9.7% engaged in sexual activity 1–3 times per month, 8.6% once a week or more, and 8.9% less than 10 times per year.

Pornography consumption over the past 7 days was also examined. While 58.5% reported not viewing pornography, 31.8% watched less than 59 minutes, 5.3% between 60 and 119 minutes, and 4.5% over 120 minutes. Further details on academic levels and departments are summarized in **Table 1**.

### 3.2 Descriptive study and normality test

The normality of the data was evaluated using skewness and kurtosis values, following the guidelines established by Mishra et al. (104). According to these guidelines, for sample sizes exceeding 300, significant deviations from normality are indicated by an absolute skewness value greater than 2 or an absolute kurtosis value greater than 7. Conversely, skewness values ≤ 2 and kurtosis values ≤ 7 suggest an acceptable approximation of normality. In this study, all variables exhibited skewness values ranging from -0.437 to 0.840 and kurtosis values ranging from -0.359 to 1.592, which fall within the acceptable limits. This confirms that the dataset meets the normality assumption, allowing parametric statistical analyses to be conducted.

All scales displayed a normal distribution, allowing parametric statistical methods to be applied. These analyses included the following constructs: ICSB_LC (representing items on lack of control), ICSB_UC (items on unwanted consequences), ICSB_NA (items on negative affect), ICSB_AR (items on affect regulation), AVS_R_T (items on traditional norms), AVS_R_F (items on freedom and flexibility), ATSPPH_SF_O (items on openness to seeking professional help), and ATSPPH_SF_N (items on the need for seeking professional help).

### 3.3 Measurement model

The data were analyzed utilizing the PLS program. The software exhibits diminished limits, as indicated by its standard distribution assumption, sample size limitations, and multicollinearity requirements. The outer loadings varied from 0.277 to 0.877 and were all statistically significant (p <.001). The components with a loading below 0.70 were retained because of the sufficient reliability of these constructs (73). **Table 2** demonstrates the reliability and validity of the endogenous latent variables. The average variance explained (AVE) for the constructs varies between 0.291 to 0.633. Cronbach’s alpha coefficients vary from 0.512 to 0.875. Cronbach’s alpha should exceed 0.70 to guarantee scale dependability; nevertheless, values below 0.70 remain acceptable (71, 72). The CR coefficients ranged from 0.706 to 0.900, beyond the 0.7 threshold (74), indicating a substantial degree of shared variance among the individual indicators. The average variance explained (AVE) for all constructs beyond 0.50; yet, we also accepted an AVE above 0.20, as an AVE below 0.50 with composite reliability exceeding 0.60 still signifies adequate convergent validity (74). The HTMT criterion is a sophisticated strategy recently introduced by Dijkstra and Henseler (75) and is extensively utilized. The discriminability of the reflective model is validated if the HTMT value for each paired construct remains below the threshold of 0.9. In the current model, the constructs of ICSB_UC and ICSB_NA demonstrated an HTMT value slightly beyond 0.9. Henseler, Ringle and Sarstedt (76) suggest that researchers must validate the confidence interval through bootstrapping techniques employed with PLS. The HTMT value is considered acceptable only when the 95% confidence interval in empirical studies excludes the value 1 and HTMT surpasses 0.9. **Table 3** presents the HTMT values.

**Table 2:** Average variance extracted, cronbach’s α, and composite reliability among the subfields of compulsive sexual behavior, Asian values, and attitudes toward seeking professional psychological help (N = 359).

Subscale	AVE	CR (rho_c)	Cronbach’s Alpha
ICSB_NA	0.633	0.873	0.806
ICSB_UC	0.532	0.900	0.875
ICSB_LC	0.542	0.889	0.870
AVS_R_T	0.291	0.838	0.792
AVS_R_F	0.305	0.830	0.805
ATSPPH_SF_N	0.331	0.706	0.512
ATSPPH_SF_O	0.540	0.851	0.793

ICSB_NA, Individual-based Compulsive Sexual Behavior – Negative Affect; ICSB_UC, Individual-based Compulsive Sexual Behavior – Unwanted consequences; ICSB_LC, Individual-based Compulsive Sexual Behavior – Lack of Control; AVS_R_T, Revision of the Asian Values – Traditional norms; AVS_R_F, Revision of the Asian Values – Freedom & Flexibility; ATSPPH_SF_N, Attitude Toward Seeking Psychology Profession Help Value and Need in Seeking Treatment; ATSPPH_SF_O, Attitudes Toward Seeking Professional Psychological Help – Openness to Seeking Help.

**Table 3:** Heterotrait-Monotrait Ratios (HTMT) of correlations among the subfields of compulsive sexual behavior, Asian values, and attitudes toward seeking professional psychological help (N = 359).

Subscale	ICSB_NA	ICSB_UC	ICSB_LC	AVS_R_T	AVS_R_F	ATSPPH_SF_N	ATSPPH_SF_O
ICSB_NA							
ICSB_UC	0.904 [0.846, 0.955]						
ICSB_LC	0.708 [0.620, 0.790]	0.821 [0.762, 0.875]					
AVS_R_T	0.352 [0.256, 0.470]	0.310 [0.253, 0.427]	0.264 [0.226, 0.374]				
AVS_R_F	0.120 [0.124, 0.223]	0.176 [0.163, 0.291]	0.125 [0.138, 0.223]	0.436 [0.405, 0.560]			
ATSPPH_SF_N	0.391 [0.278, 0.540]	0.383 [0.284, 0.532]	0.430 [0.319, 0.575]	0.422 [0.361, 0.637]	0.414 [0.366, 0.596]		
ATSPPH_SF_O	0.180 [0.115, 0.310]	0.129 [0.105, 0.248]	0.207 [0.150, 0.329]	0.301 [0.236, 0.453]	0.340 [0.283, 0.469]	0.501 [0.406, 0.682]	

ICSB_NA, Individual-based Compulsive Sexual Behavior – Negative Affect; ICSB_UC, Individual-based Compulsive Sexual Behavior – Unwanted consequences; ICSB_LC, Individual-based Compulsive Sexual Behavior – Lack of Control; AVS_R_T, Revision of the Asian Values – Traditional norms; AVS_R_F, Revision of the Asian Values – Freedom & Flexibility; ATSPPH_SF_N, Attitude Toward Seeking Psychology Profession Help Value and Need in Seeking Treatment; ATSPPH_SF_O, Attitudes Toward Seeking Professional Psychological Help – Openness to Seeking Help.

### 3.4 Structural model

This study reports total effects to capture the overall influence of predictors on the outcome variables, while indirect effects are presented to examine mediation pathways. Prior to assessing the structural model, it is imperative to address the collinearity issue to mitigate bias in the regression outcomes. The Variance Inflation Factors (VIFs) in the model ranged from 1.197 to 3.948, constantly remaining under the threshold of 5.0 (77). Thus, we may conclude that collinearity is not an issue in the current model (**Table 4**).

**Table 4:** Collinearity statistics.

	VIF
AVS_R_F -> ATSPPH_SF_O	1.611
AVS_R_T -> ATSPPH_SF_N	1.197
ICSB_LC -> ATSPPH_SF_O	2.655
ICSB_LC -> AVS_R_F	2.686
ICSB_NA -> ATSPPH_SF_N	1.451
ICSB_NA -> AVS_R_F	2.913
ICSB_NA -> AVS_R_T	1.336
ICSB_UC -> ATSPPH_SF_O	3.296
ICSB_UC -> AVS_R_F	3.948

ICSB_NA, Individual-based Compulsive Sexual Behavior – Negative Affect; ICSB_UC, Individual-based Compulsive Sexual Behavior – Unwanted consequences; ICSB_LC, Individual-based Compulsive Sexual Behavior – Lack of Control; AVS_R_T, Revision of the Asian Values – Traditional norms; AVS_R_F, Revision of the Asian Values – Freedom & Flexibility; ATSPPH_SF_N, Attitude Toward Seeking Psychology Profession Help Value and Need in Seeking Treatment; ATSPPH_SF_O, Attitudes Toward Seeking Professional Psychological Help – Openness to Seeking Help.

**Figure 1:**
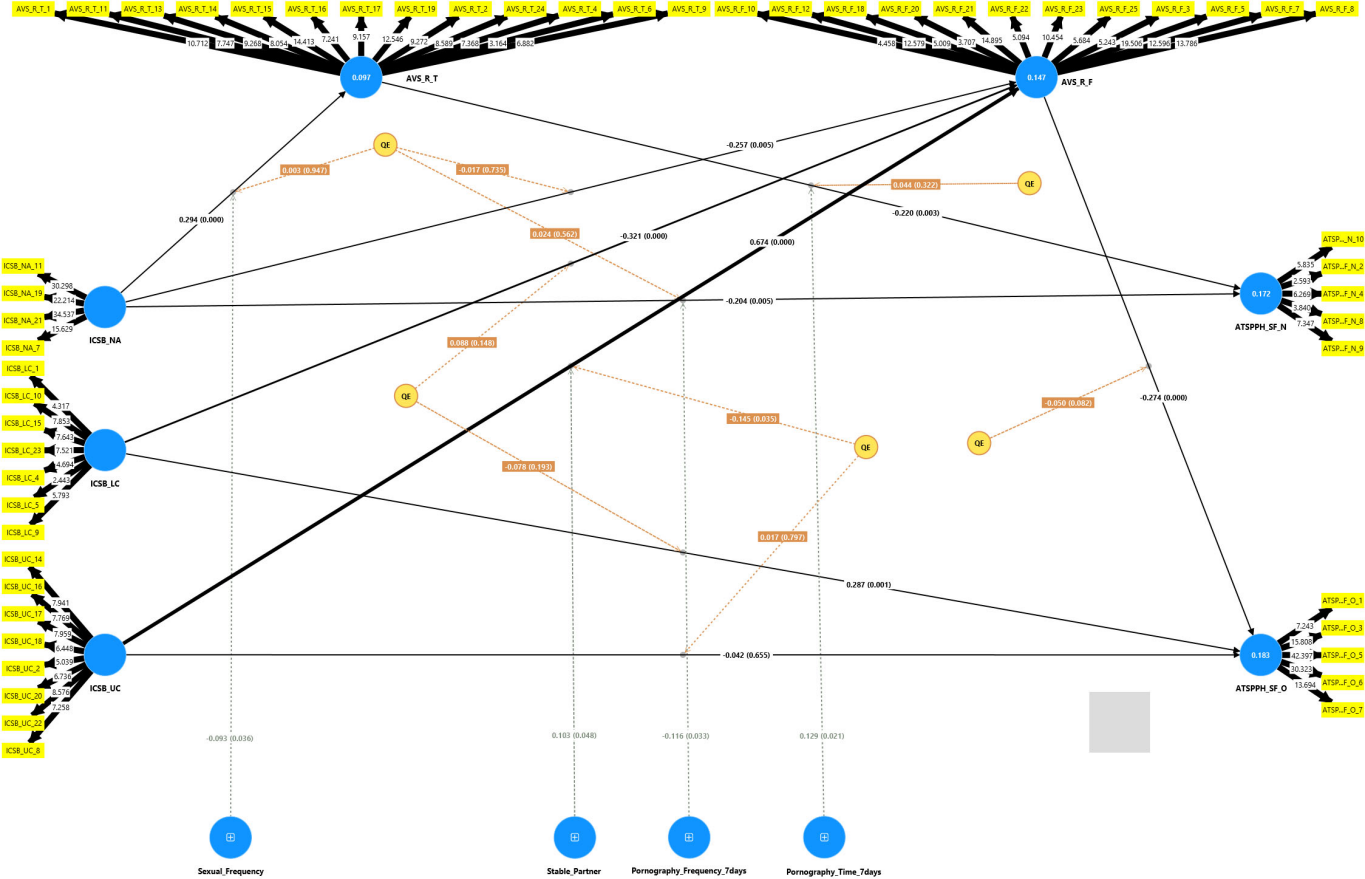
Partial least squares structural equation modeling (PLS-SEM) results. ICSB_NA, Individual-based Compulsive Sexual Behavior – Negative Affect; ICSB_UC, Individual-based Compulsive Sexual Behavior – Unwanted consequences; ICSB_LC, Individual-based Compulsive Sexual Behavior – Lack of Control; AVS_R_T, Revision of the Asian Values – Traditional norms; AVS_R_F, Revision of the Asian Values – Freedom & Flexibility; ATSPPH_SF_N, Attitude Toward Seeking Psychology Profession Help Value and Need in Seeking Treatment; ATSPPH_SF_O, Attitudes Toward Seeking Professional Psychological Help – Openness to Seeking Help.

**Figure 1** displayed the path coefficients, which elucidate the correlations among the variables.

The findings show that lack of control in CSB negatively impacts on freedom and flexibility of Asian values (β = -0.321, 95% CI [0.469, -0.114], *p* <.001). The negative affect of CSB negatively impacts on freedom and flexibility of Asian values (β = -0.257, 95% CI [-0.418, -0.056], *p* < 0.05). Additionally, negative affect was also found to have a positive impact on traditional values (β = 0.294, 95% CI [0.196, 0.425], *p* <.001). Besides, unwanted consequences in CSB positively influence freedom and flexibility values (β = 0.674, 95% CI [0.361, 0.849], *p* <.001). *From these results, the first hypothesis (H1) was confirmed*. The results indicate that the quadratic effect (QE) of unwanted consequences on freedom and flexibility values was significant (β = -0.145, 95% CI [-0.273, -0.001], *p* < 0.05), suggesting an inverted U-shaped relationship. This implies that as unwanted consequences increase, its effect on freedom and flexibility values changes direction at a certain threshold.

On the other hand, freedom and flexibility values were found to have a negative effect on openness to seeking professional psychological help (β = -0.274, 95% CI [-0.401, -0.162], *p* <.001). Similarly, the traditional values also negatively impact the needs in seeking professional psychological help (β = -0.220, 95% CI [0.378, - 0.091], *p* < 0.05). *The second hypothesis (H2) was validated*.

Negative affect had a negative impact on the needs in seeking professional psychological help (β = -0.269, 95% CI [-0.392, -0.141], *p* <.001) but it had a positively effect on openness to seeking professional psychological help (β = 0.071, 95% CI [0.016, 0.134], *p* < 0.05). Furthermore, lack of control in CSB positively influenced openness to seeking professional psychological help (β = 0.375, 95% CI [0.178, 0.535], *p* <.001), and unwanted consequences, in turn, had a negative impact on openness to seeking professional psychological help (β = -0.227, 95% CI [-0.413, -0.001], *p* < 0.05). *The third hypothesis (H3) was verified.*

The effect size was greatest for the pathway from negative affect in CSB to freedom and flexibility of Asian values (f^2^ = 0.135, *p* < 0.05), signifying a medium-level influence (74, 75). The R^2^ for the variance predicted in the endogenous constructs ranged from 9.7% to 18.3%. The corrected R² varied between 8.6% and 16.9%, signifying a low degree of predictive accuracy (76, 77). The model constructs had significant predictive power, evidenced by Q^2^ predict values over zero, ranging from 0.002 to 0.093.

The direct and indirect effects can be found in **Table 5**.

**Table 5:** The mediating effect of Asian values and the moderate role of sexual frequency, having stable sexual partner, frequency of watching pornography (in a week), times of watching pornography (in a week) (N = 359).

Hyp.	Path	β	Confidence intervals 95%	t	p
Direct effect					
H1	ICSB → AVS				
H1.1	ICSB_LC → AVS_R_F	-0.321	[0.469, -0.114]	3.543	<.001
H1.2	ICSB_NA → AVS_R_F	-0.257	[-0.418, -0.056]	2.810	0.005
H1.3	ICSB_NA → AVS_R_T	0.294	[0.196, 0.425]	4.999	<.001
H1.4	ICSB_UC → AVS_R_F	0.674	[0.361, 0.849]	4.963	<.001
H2	AVS → ATSPPH				
H2.1	AVS_R_F → ATSPPH_SF_O	-0.274	[-0.401, -0.162]	4.464	<.001
H2.2	AVS_R_T → ATSPPH_SF_N	-0.220	[0.378, - 0.091]	2.987	0.003
H3	ICSB → ATSPPH				
H3.1	ICSB_LC → ATSPPH_SF_O	0.375	[0.178, 0.535]	3.816	<.001
H3.2	ICSB_NA → ATSPPH_SF_N	-0.269	[-0.392, -0.141]	4.174	<.001
H3.3	ICSB_NA → ATSPPH_SF_O	0.071	[0.016, 0.134]	2.311	0.021
H3.4	ICSB_UC → ATSPPH_SF_O	-0.227	[-0.413, -0.001]	2.168	0.030
H4	Pornography_Frequency_7days x ICSB_NA → ATSPPH_SF_N	-0.116	[-0.214, 0.002]	2.132	0.033
H5	Pornography_Time_7days x AVS_R_T → ATSPPH_SF_N	0.129	[0.014, 0.230]	2.317	0.021
H6	Sexual_Frequency x ICSB_NA → AVS_R_T	-0.093	[-0.171, 0.003]	2.095	0.036
H7	Stable_Partner x ICSB_UC → AVS_R_F	0.103	[-0.000, 0.203]	1.979	0.048
Indirect effect					
H8	ICSB → AVS → ATSPPH				
H8.1	ICSB_NA → AVS_R_T → ATSPPH_SF_N	-0.065	[-0.133, -0.026]	2.370	0.018
H8.2	ICSB_LC → AVS_R_F → ATSPPH_SF_O	0.088	[0.029, 0.147]	2.895	0.004
H8.3	ICSB_NA → AVS_R_F → ATSPPH_SF_O	0.071	[0.016, 0.134]	2.311	0.021
H8.4	ICSB_UC → AVS_R_F → ATSPPH_SF_O	-0.185	[-0.280, -0.084]	3.530	<.001

ICSB_NA, Individual-based Compulsive Sexual Behavior – Negative Affect; ICSB_UC, Individual-based Compulsive Sexual Behavior – Unwanted consequences; ICSB_LC, Individual-based Compulsive Sexual Behavior – Lack of Control; AVS_R_T, Revision of the Asian Values – Traditional norms; AVS_R_F, Revision of the Asian Values – Freedom & Flexibility; ATSPPH_SF_N, Attitude Toward Seeking Psychology Profession Help Value and Need in Seeking Treatment; ATSPPH_SF_O, Attitudes Toward Seeking Professional Psychological Help – Openness to Seeking Help.

### 3.5 Mediation and moderation analysis

The study revealed a significant indirect effect of negative affect of CSB on needs in seeking professional psychological help, mediated by tradition values (β = -0.065, 95% CI [-0.133, -0.026], *p* < 0.05). Freedom and flexibility in Asian values also played mediation role between lack of control in CSB and openness to seeking professional psychological help (β = 0.088, 95% CI [0.029, 0.147], *p* < 0.05); negative affect in CSB and openness to seeking professional psychological help (β = 0.071, 95% CI [0.016, 0.134], *p* < 0.05); unwanted consequences in CSB and openness to seeking professional psychological help (β = -0.185, 95% CI [-0.280, -0.084], *p* <.001). *From these result, the eighth hypothesis (H8) was affirmed*. Additionally, *hypothesis 4 was supported*, indicating that the frequency of watching pornography negatively moderated the association between negative affect in CSB and the need to seek professional psychological help (β = -0.116, 95% CI [-0.214, 0.002], *p* < 0.05). Moreover, times watching pornography also moderated the relationship between traditional values and needs in seeking professional psychological help (β = 0.129, 95% CI [0.014, 0.230], *p* < 0.05). The frequency of having sex impacted the relation between negative affect and traditional values (β = -0.093, 95% CI [-0.171, 0.003], *p* < 0.05). Finally, having a stable sexual partner played a moderator role between unwanted consequences in CSB and freedom and flexibility values (β = 0.103, 95% CI [-0.000, 0.203], *p* < 0.05). In the results, *the fifth hypothesis (H5), the sixth hypothesis (H6), and the seventh hypothesis (H7) were validated* (see in **Table 5**).

## 4 Discussion

The study objective to examine the relationship between compulsive sexual behavior (CSB), Asian values (including the endorsement of freedom and flexibility in values and adherence to traditional Asian values), and attitudes toward seeking professional psychological help. Additionally, it explored the moderating effects of pornography consumption, sexual frequency, and having a stable sexual partner on this relationship. Furthermore, the study explored how Asian values relate to CSB and openness to seeking professional psychological help, specifically focusing on the mediating role of Asian values in the relationship between negative affect or unwanted consequences of CSB and the tendency to seek psychological support. These findings offer valuable insights into how Asian values shape individuals’ experiences of CSB and influence their willingness to seek help, contributing to more effective clinical interventions and mental health support strategies.

### 4.1 The relationship between CSB and Asian values

The research findings demonstrated a strong association between CSB and Asian cultural values (H1). Specifically, the level of lack of control (H1.1) and the negative affect of CSB are negatively associated with the degree of freedom and flexibility within Asian values (H1.2). In addition, the study has shown that the negative affect of CSB was positively associated with traditional cultural values in Asian societies (H1.3). In other words, individuals who struggled to regulate CSB and experienced greater negative affect from this behavior tended to maintain less flexible and less liberal value systems, or adhered more strongly to traditional cultural norms (78, 79). These findings suggest that internal conflicts between behaviors and personal values may reinforce rigid value systems (80). Moreover, psychological rigidity has been associated with avoidance behaviors, which further strengthen cognitive inflexibility (81). These results are consistent with the theory of cognitive rigidity, which posits that emotional distress reinforces rigid thinking patterns and limits one’s adaptive capacity (78, 82). Individuals experiencing negative emotions related to CSB may have internalized guilt and self-stigma, thereby reinforcing their adherence to traditional values in an effort to preserve moral and social conformity (83, 84).

However, the findings also revealed that individuals who experienced undesirable consequences from CSB tended to exhibit greater openness and a reduced adherence to traditional Asian values (H1.4). This phenomenon can be explained through Janoff-Bulman’s Shattered Assumptions Theory (1992). When confronted with the distressing consequences of CSB, individuals may have encountered psychologically disruptive experiences that directly challenged their traditional beliefs and moral frameworks. As a result, such individuals experienced psychological dissonance that compelled them to restructure their value systems in more flexible and adaptive ways (85).

### 4.2 The relationship between Asian values and attitude toward seeking professional psychological help

The findings showed that individuals who endorsed greater freedom and flexibility in Asian cultural values tended to exhibit lower levels of openness toward seeking professional psychological help (H2.1). This result contradicts the findings of Shea and Yeh (49), who suggested that individuals with more flexible value systems may be more willing to seek help due to being less constrained by cultural stigma surrounding mental health care. Although such individuals hold more liberal perspectives on traditional values, they may also perceive mental health challenges as personal matters that should be managed independently, which could reduce their likelihood of seeking external assistance (86).

In addition, individuals who adhered more strongly to traditional cultural values were found to be less likely to seek professional help, whereas those with lower adherence to traditional values tended to be more open to accessing psychological support (H2.2). This finding aligns with prior research indicating that those who strictly follow traditional cultural norms may perceive psychological distress as a private burden that should be resolved independently rather than through professional intervention (43–47). This pattern is consistent with broader literature emphasizing that, within Asian cultures, beliefs regarding help-seeking – including attitudes toward mental illness, personal responsibility, and stigma associated with therapy – contribute to the perception that mental health struggles are private concerns to be dealt with individually (39–46).

### 4.3 The relationship between CSB and attitudes toward seeking professional psychological help

The present study found that a lack of control over CSB was positively associated with openness to seeking professional psychological help (H3.1). This finding aligns with the Theory of Planned Behavior, which suggests that individuals with lower perceived behavioral control are more likely to seek external assistance to manage their difficulties (87). When individuals perceive their CSB as unmanageable, they may become more willing to engage with professional support services (88). Prior research has shown that uncontrolled CSB contributes to significant emotional distress, increasing the likelihood of seeking psychological help (89). Individuals experiencing severe distress due to CSB often report higher levels of depression, anxiety, and impaired social functioning, which can act as catalysts for help-seeking behaviors (90). Moreover, those who recognize the severity of their symptoms tend to be more proactive in accessing mental health resources (91). These findings highlight the need for interventions that enhance awareness of CSB-related distress and promote access to professional psychological services. Future research should explore the role of self-efficacy and perceived behavioral control in facilitating help-seeking behaviors among individuals struggling with CSB.

Our findings presented a nuanced and seemingly contradictory role for negative affect in CSB. The findings found that negative affect resulting from CSB is positively associated with openness to seek professional psychological help (H3.3). Specifically, individuals experiencing greater emotional distress due to CSB demonstrate a higher willingness to pursue psychological assistance, consistent with prior research suggesting that psychological discomfort motivates mental health help-seeking (92). These findings align with the Health Belief Model (93), which suggests that individuals are more likely to seek help when perceiving a significant personal threat. These findings collectively underscore the need for interventions that enhance awareness of CSB-related distress and promote access to professional psychological services. Future research should explore the roles of self-efficacy and perceived behavioral control in facilitating help-seeking behaviors among individuals struggling with CSB.

On the other hand, the same negative affect resulting from CSB was negatively associated with need to seeking professional psychological help (H3.2). This apparent paradox highlights a critical distinction between an individual’s willingness to consider help (openness) and their internal recognition of its necessity (need). While distress may create a general awareness of the problem, it does not automatically translate into a personal conviction that professional help is required. Prior research indicates that heightened psychological distress often reinforces avoidance tendencies, leading individuals to withdraw rather than seek external assistance (44). Self-stigmatization plays a crucial role in reducing help-seeking behaviors, as individuals struggling with CSB may fear social judgment or internalize shame, thereby minimizing their perceived need for therapy (94). Moreover, cultural influences can exacerbate this avoidance, particularly in societies where sexual behavior is heavily moralized, leading to increased reluctance to acknowledge mental health needs (95).

Notably, the study found that unwanted consequences of CSB are negatively associated with openness to seeking professional psychological help (H3.4). Individuals experiencing greater negative repercussions from CSB appear less inclined to seek assistance, which aligns with research on help-seeking barriers in sexual behaviors (88, 96). One explanation for this relationship is the role of shame and self-stigma, which have been shown to deter individuals from pursuing psychological support (97). Additionally, avoidance coping strategies, such as denial and minimization, may further reduce help-seeking tendencies, as individuals attempt to manage distress without external intervention (94). Some may also perceive psychological treatment as ineffective or unnecessary, leading them to rely on self-directed coping strategies instead (96). The fear of social judgment and cultural stigma surrounding CSB may further discourage individuals from seeking professional help (98).

### 4.4 Moderating roles of pornography consumption in CSB, Asian values, and help-seeking attitudes

The findings demonstrated that the frequency of pornography consumption per week moderated the relationship between the negative affect of CSB and the intention to seek professional help (H4). This suggests that frequent exposure to pornographic content may either normalize the discomfort associated with CSB or intensify feelings of shame, thereby diminishing the motivation to pursue psychological support (26). One possible explanation is that frequent pornography use may reinforce compulsive patterns of behavior and reduce individuals’ ability to recognize problematic symptoms (99). Moreover, repeated exposure to pornographic material may desensitize individuals’ emotional responses, making them less likely to perceive their psychological distress as something requiring professional intervention (94). These findings underscore the complex role of pornography use in shaping individuals’ awareness of psychological difficulties and their willingness to seek help, highlighting the need for more nuanced approaches in therapeutic settings targeting individuals with CSB.

The results showed that the amount of time spent viewing pornography per week moderated the relationship between adherence to traditional Asian cultural values and the intention to seek psychological help (H5). Specifically, pornography consumption weakens the negative relationship between adherence to traditional Asian values and the intention to seek psychological help. This pattern may reflect underlying cognitive dissonance, as individuals who uphold traditional values may experience psychological distress when engaging in behaviors that conflict with their moral or cultural beliefs (94). Prolonged exposure to pornographic content may challenge rigid sexual norms and trigger internal conflict, which in turn affects how individuals perceive their mental health needs and their willingness to seek help (8). These findings suggest that the intersection between cultural values and media consumption plays a critical role in shaping psychological help-seeking behavior. In contexts where traditional moral frameworks dominate, high levels of pornography use may further complicate the emotional and cognitive processes involved in recognizing the need for intervention.

The findings indicated that the frequency of sexual activity moderated the relationship between the negative affect of CSB and adherence to traditional Asian cultural values (H6). Specifically, the positive association between the adverse consequences of CSB and the tendency to uphold traditional cultural values was weakened at higher frequencies of sexual activity. This pattern may reflect the possibility that individuals turn to traditional values as a coping mechanism to protect their sense of identity and meaning in life when experiencing psychological conflict (85). This finding aligns with the argument that regular sexual engagement may reduce the need to reaffirm traditional moral beliefs (94). Conversely, individuals with lower levels of sexual activity may experience greater cognitive dissonance and, as a result, be more likely to reinforce traditional values as a way to manage psychological distress (94). At the same time, prior research has suggested that individuals who are more sexually active tend to report lower levels of sexual guilt and are more accepting of non-traditional sexual norms (26). Therefore, the frequency of sexual behavior may serve as both a behavioral and psychological moderator that influences how individuals reconcile the distress caused by CSB with their underlying cultural belief systems.

The findings revealed that having stable sexual partner moderated the relationship between the unwanted consequences of CSB and adherence to more liberal and flexible Asian cultural values (H7). Specifically, in cases where the negative impacts of CSB increased, the presence of a stable romantic relationship appeared to weaken the association between those impacts and the endorsement of rigid traditional values. In other words, individuals with a committed partner tended to adopt a more flexible and liberal stance toward their cultural value systems. This finding may reflect the psychological buffering role of long-term romantic relationships, which can provide emotional support that helps individuals regulate internal distress and reduce the need to rely on rigid cultural norms as a coping strategy (e.g., Janoff-Bulman, 1992 (85). The emotional security and mutual understanding offered by a committed relationship may facilitate a more adaptive and less defensive engagement with cultural expectations, especially in the context of psychological conflict related to CSB.

### 4.5 Indirect effect of CSB on help-seeking through Asian values

The present study found that the indirect effect of negative affect resulting from CSB on the perceived need for professional help through adherence to traditional values. Individuals experiencing heightened distress from CSB tend to reinforce traditional values, which in turn lowers their likelihood of seeking psychological assistance (67). This may be explained by cognitive dissonance theory (100), which suggests that adherence to traditional values serves as a coping mechanism to reduce moral conflict while discouraging help-seeking due to stigma (101). Traditional values emphasize resilience and self-reliance, leading individuals to rely on informal support rather than professional services (89). Additionally, self-stigma may reinforce the belief that psychological help is unnecessary, further reducing help-seeking intentions (102).

The present study found that the indirect effect of a lack of control over CSB on openness to seeking professional psychological help through freedom and flexibility in values (H8.2). Specifically, individuals experiencing greater difficulties in controlling their CSB tend to have lower value flexibility, which, in turn, increases their willingness to seek psychological assistance. This finding aligns with research suggesting that distress can lead to psychological rigidity, limiting adaptive coping strategies (81). When rigid coping fails to alleviate distress, individuals may recognize professional help as a necessary alternative (90). Additionally, perceived loss of control over behavior is associated with lower self-efficacy, increasing the likelihood of seeking external support (91).

The results revealed a positive indirect relationship from negative affect associated with CSB to openness toward seeking professional psychological help mediated by the endorsement of freedom and flexibility within Asian cultural values. Specifically, higher levels of negative affect resulting from compulsive sexual behavior were associated with lower levels of endorsement of freedom-flexibility values. In turn, lower levels of freedom-flexibility were linked to greater openness to seeking psychological help. As previously mentioned, this finding is consistent with earlier studies (78, 80), which suggested that individuals experiencing high levels of emotional distress related to CSB tend to be less flexible in their cultural value orientations. Notably, the finding that lower levels of freedom-flexibility predict greater help-seeking openness contrasts with prior research (e.g (e.g., (49)), which generally posits that more open and flexible cultural values are associated with more positive attitudes toward seeking professional help. At face value, this also appears to contradict the results of Hypothesis H2.2 in the present study.

However, several explanations may account for this phenomenon. Firstly, the findings indicate that both traditional values and freedom-flexibility values are negatively associated with help-seeking attitudes. This may reflect a value conflict or crisis among today’s youth, who are simultaneously influenced by both traditional and modern value systems. When taken to extremes or misinterpreted, either value orientation may hinder help-seeking behavior, those with strong traditional values may avoid seeking help to preserve face, while those with high autonomy values may prefer self-reliance or fear being judged (103). Secondly, the experience of intense negative emotions stemming from compulsive sexual behavior may alter how individuals interpret cultural values. In moments of psychological distress, values such as freedom and flexibility may no longer be perceived as empowering, but rather as sources of internal conflict or instability, thereby prompting individuals to seek external support. Lastly, the characteristics of the sample may reflect a population in psychological crisis, with heightened vulnerability and emotional urgency. These individuals may be driven to seek professional, help not out of a consistent endorsement of individual freedom, but from a need for stability and containment. In such cases, lower levels of freedom-flexibility may coincide with a greater readiness to engage in professional help-seeking. Taken together, these interpretations may represent a novel contribution of the current study, offering deeper insights into how cultural values interact with emotional distress to shape help-seeking tendencies.

The present study found that freedom and flexibility in values mediate the association between the unwanted consequences of CSB and openness to professional help-seeking (H8.4). Specifically, individuals experiencing distress from CSB might undergo a cognitive reevaluation of their values, shifting from rigid to more flexible cultural perspectives. However, this shift does not necessarily promote help-seeking. The adoption of flexible values may reflect a compensatory coping strategy or an attempt to distance oneself from socially imposed norms and stigma, which can ironically reduce the likelihood of seeking formal psychological support (103). As suggested by Leong, Kim and Gupta (95), culturally responsive approaches that balance traditional and modern values may enhance help-seeking attitudes, but only when accompanied by emotional safety and reduced stigma. Future research could explore the qualitative meanings behind value change to better inform interventions in contexts involving sexual distress.

### 4.6 Implications

The findings of this study offer important implications for both clinical practice and mental health interventions, particularly in cultural contexts that emphasize traditional Asian values. The results suggest that Asian values play a crucial role in shaping individuals’ experiences of compulsive sexual behavior (CSB) and their openness to seeking professional psychological help. Given the influence of traditional values on help-seeking attitudes, mental health professionals should adopt culturally sensitive approaches that acknowledge the role of familial expectations, social harmony, and personal responsibility in shaping individuals’ perceptions of psychological distress and treatment.

The moderating role of having a stable sexual partner highlights the importance of relationship dynamics in coping with CSB-related distress. Interventions should consider how intimate relationships provide emotional and psychological support, potentially fostering greater value flexibility and reducing stigma around seeking professional help. Moreover, the study underscores the need for therapeutic strategies that enhance psychological flexibility, such as Acceptance and Commitment Therapy (ACT) and Cognitive Behavioral Therapy (CBT), to help individuals navigate value conflicts and maladaptive coping mechanisms associated with CSB.

Additionally, public health efforts should focus on reducing stigma surrounding CSB and mental health treatment, particularly in collectivist cultures where shame and self-stigma may act as barriers to help-seeking. Psychoeducation programs tailored to Asian cultural values could help normalize discussions around sexual health and encourage individuals to seek appropriate support without fear of moral judgment.

Future research should further investigate how cultural factors, including religiosity, intergenerational value transmission, and social expectations, interact with CSB and mental health help-seeking behaviors. Understanding these dynamics will contribute to more effective, culturally responsive mental health interventions that support individuals struggling with CSB while respecting their value systems.

### 4.7 Limitations and future directions

Although this study provided important contributions, several limitations should be considered. First, the cross-sectional design did not allow for establishing causal relationships between the studied variables. Future research should consider using a longitudinal approach to examine these relationships over time.

Second, the sample primarily consisted of university students in Ho Chi Minh City, which may limit the generalizability of the findings to other populations. Future studies should expand the sample to include individuals from diverse age groups and cultural backgrounds to enhance the representativeness of the results. Additionally, incorporating more objective measurement methods could help minimize biases associated with self-report data.

Third, the reliability coefficient (Cronbach’s Alpha) for the Attitudes Toward Seeking Professional Psychological Help Scale did not reach an optimal level, potentially affecting the accuracy of assessing attitudes toward seeking professional psychological help. Nonetheless, the relatively low reliability of the overall scale and particularly of the openness-to-support subscale, means the authors must interpret any group differences or correlational findings with caution. In practical terms, measurement error introduced by lower internal consistency can attenuate observed effect sizes and reduce statistical power, making it harder to detect true associations between attitudes and help-seeking behaviors. Consequently, any non-significant or modest associations involving the ATSPPH-SF may partly reflect instrument imprecision rather than an absence of substantive relationships. Future research should consider utilizing more reliable scales or refining the measurement tool to ensure greater accuracy in assessment.

## 5 Conclusion

This study examined the relationship between compulsive sexual behavior (CSB), Asian values, and openness to seeking professional psychological help within an Asian cultural context. The findings highlighted the role of adherence to traditional values and flexibility in shaping help-seeking attitudes, with sexual frequency and having a stable sexual partner acting as moderating factors. By identifying Asian values as key mediators, this research provided insights for culturally sensitive interventions addressing stigma and value-related barriers to psychological support. Mental health professionals should consider strategies that enhance psychological flexibility while respecting cultural beliefs to promote help-seeking behavior. Future research should explore additional cultural influences, such as family expectations and religious values, to refine mental health interventions. These findings contribute to a deeper understanding of the psychological and social dynamics influencing CSB, informing more inclusive and effective support strategies.

## Data Availability

The raw data supporting the conclusions of this article will be made available by the authors, without undue reservation.
