# Supplementary material for: Asian cultural values and help-seeking: a cross-sectional study on compulsive sexual behavior
Source: Front Psychiatry. 2025 Oct 7;16:1633160. doi: 10.3389/fpsyt.2025.1633160 (PMC12538654; doi:10.3389/fpsyt.2025.1633160)
Supplement: Supplementary file 1 [file DataSheet1.docx]

Supplementary Material

# Supplementary Figures and Tables

## Supplementary Figures


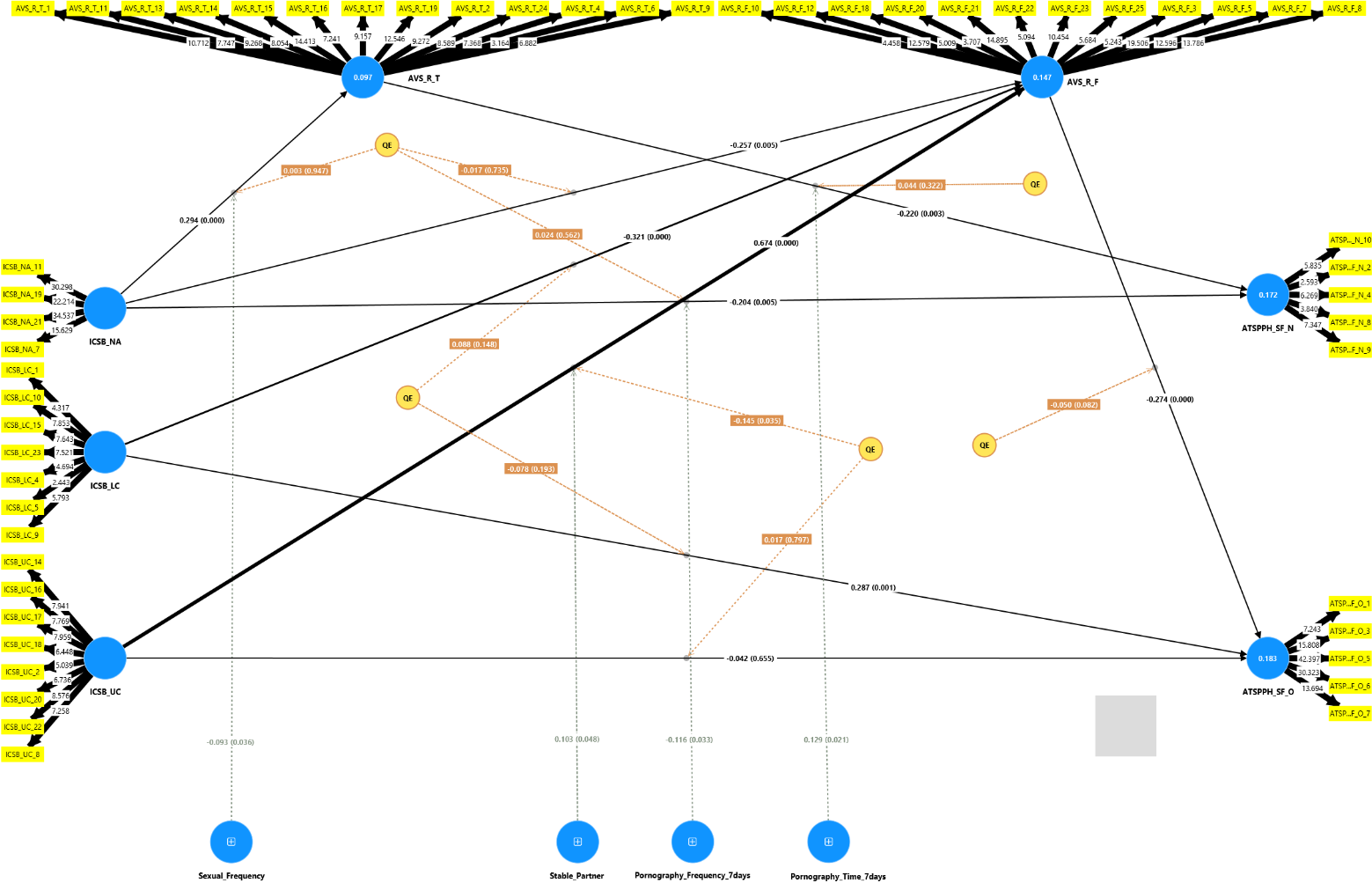


**Supplementary Figure 1.** Partial least squares structural equation modeling (PLS-SEM) results

**Abbreviations:** ICSB_NA Individual-based Compulsive Sexual Behavior - Negative Affect, ICSB_UC Individual-based Compulsive Sexual Behavior- Unwanted consequences, ICSB_LC Individual-based Compulsive Sexual Behavior - Lack of Control, AVS_R_T Revision of the Asian Values- Traditional norms, AVS_R_F Revision of the Asian Values- Freedom & Flexibility, ATSPPH_SF_N Attitude Toward Seeking Psychology Profession Help Value and Need in Seeking Treatment, ATSPPH_SF_ O Attitudes Toward Seeking Professional Psychological Help - Openness to Seeking Help.

## Supplementary Table

**Table 1** Demographic characteristics

| **Variable** | | **Total**  **(n= 359)**  **Frequency (%)** | **ICSB**  **(n=359)**  **Mean ± SD** | ***AV***  ***(n=359)***  ***Mean ± SD*** | ***ATSPPH***  ***(n=359)***  ***Mean ± SD*** |
| --- | --- | --- | --- | --- | --- |
| Gender | Male | 135 (37.6) | 3.06 ± 1.2 | 2.45 ± 0.25 | 2.99 ± 0.49 |
|  | Female | 224 (62.4) | 2.31 ± 1.01 | 2.31 ± 0.28 | 3.20 ± 0.43 |
| Sexual Orientation | Heterosexual | 212 (59.1) | 2.53 ± 1.08 | 2.39 ± 0.25 | 3.12 ± 0.48 |
|  | LGBTQ+ | 147 (40.9) | 2.67 ± 1.23 | 2.33 ± 0.29 | 3.13 ± 0.44 |
| Academic Levels | Freshman | 123 (34.3) | 2.43 ±  1.17 | 2.39 ± 0.26 | 3.04 ± 0.46 |
|  | Sophomore | 45 (12.5) | 2.90 ± 1.33 | 2.38 ± 0.25 | 3.19 ± 0.45 |
|  | Junior | 118 (32.9) | 2.64 ± 1.13 | 2.34 ± 0.27 | 3.14 ± 0.46 |
|  | Senior or above | 73 (20.3) | 2.61 ± 0.95 | 2.34 ± 0.31 | 3.18 ± 0.47 |
| Academic field | Education | 59 (16.4) | 2.43 ± 1.20 | 2.39 ± 0.25 | 3.11 ± 0.40 |
|  | Humanities | 161 (44.8) | 2.63 ± 1.13 | 2.31 ± 0.28 | 3.20 ± 0.50 |
|  | Business | 37 (10.3) | 2.71 ± 1.15 | 2.36 ± 0.31 | 2.98 ± 0.42 |
|  | Natural sciences | 25 (7.0) | 2.65 ± 1.19 | 2.44 ± 0.25 | 3.02 ± 0.42 |
|  | Engineering & Technology | 61 (17.0) | 2.57 ± 1.12 | 2.43 ± 0.25 | 3.06 ± 0.44 |
|  | Other | 16 (4.5) | 2.51 ± 1.07 | 2.48 ± 0.21 | 3.05 ± 0.49 |
| Area | Urban area | 285 (79.4) | 2.59 ±  1.12 | 2.35 ± 0.29 | 3.12 ± 0.48 |
|  | Rural area | 74 (20.6) | 2.60 ±  1.23 | 2.41 ± 0.21 | 3.10 ± 0.40 |
| Religion | No religion/belief | 158 (44.0) | 2.45 ± 1.06 | 2.37 ± 0.28 | 3.07 ± 0.52 |
|  | Practice of religion/ belief | 67 (18.7) | 2.86 ± 1.26 | 2.42 ± 0.26 | 3.16 ± 0.42 |
|  | No practice but belief | 134 (37.3) | 2.63 ± 1.17 | 2.33 ± 0.27 | 3.17 ± 0.43 |
| Stable Partner | Not sexually active | 258 (71.9) | 2.51 ± 1.10 | 2.37 ± 0.27 | 3.12 ± 0.47 |
|  | Have a stable sexual partner | 59 (16.4) | 2.91 ± 1.18 | 2.35 ± 0.25 | 3.10 ± 0.49 |
|  | Do not have a stable sexual partner | 42 (11.7) | 2.61 ± 1.28 | 2.37 ± 0.30 | 3.13 ± 0.39 |
| Sexual Frequency | Never had sexual activity | 261 (72.7) | 2.48 ± 1.12 | 2.37 ± 0.27 | 3.13 ± 0.47 |
|  | Less than 10 times a year | 32 (8.9) | 2.91 ± 1.01 | 2.35 ± 0.31 | 3.11 ± 0.43 |
|  | 1-3 times per month | 35 (9.7) | 2.81 ± 1.02 | 2.31 ± 0.25 | 3.15 ± 0.38 |
|  | Once a week or more | 31 (8.6) | 2.94 ± 1.45 | 2.39 ± 0.25 | 3.05 ± 0.54 |
| Pornography Time 7 days | None | 210 (58.5) | 2.30 ± 0.99 | 2.37 ± 0.27 | 3.14 ± 0.43 |
|  | 59 minutes or less | 114 (31.8) | 2.79 ± 1.16 | 2.36 ± 0.27 | 3.19 ± 0.43 |
|  | 60-119 minutes | 19 (5.3) | 3.39 ± 0.83 | 2.26 ± 0.32 | 2.77 ± 0.54 |
|  | 120 minutes and more | 16 (4.5) | 4.08 ± 1.32 | 2.52 ± 0.21 | 2.80 ± 0.72 |
| Pornography Frequency 7 days | None | 215 (59.9) | 2.31 ± 0.99 | 2.37 ± 0.28 | 3.15 ± 0.43 |
|  | 1-3 times | 107 (29.8) | 2.78 ± 1.12 | 2.35 ± 0.29 | 3.12 ± 0.49 |
|  | 4-6 times | 20 (5.6) | 3.51 ± 1.02 | 2.39 ± 0.20 | 2.95 ± 0.50 |
|  | More than 6 times | 17 (4.7) | 3.91 ± 1.51 | 2.41 ± 0.24 | 2.92 ± 0.64 |

**Note:** **<0.05; ***< 0.001

**Abbreviations:** ICSB Individual Compulsive Sexual Behavior; AV Asian Values; ATSPPH Attitude Toward Seeking Psychology Profession Help.

**Table 2** Average variance extracted, cronbach’s α, and composite reliability among the subfields of compulsive sexual behavior, Asian values, and attitudes toward seeking professional psychological help (N = 359)

| ***Subscale*** | ***AVE*** | ***CR (rho_c)*** | ***Cronbach’s Alpha*** |
| --- | --- | --- | --- |
| ICSB_NA | 0.633 | 0.873 | 0.806 |
| ICSB_UC | 0.532 | 0.900 | 0.875 |
| ICSB_LC | 0.542 | 0.889 | 0.870 |
| AVS_R_T | 0.291 | 0.838 | 0.792 |
| AVS_R_F | 0.305 | 0.830 | 0.805 |
| ATSPPH_SF_N | 0.331 | 0.706 | 0.512 |
| ATSPPH_SF_O | 0.540 | 0.851 | 0.793 |

**Abbreviations:** ICSB_NA Individual-based Compulsive Sexual Behavior - Negative Affect, ICSB_UC Individual-based Compulsive Sexual Behavior- Unwanted consequences, ICSB_LC Individual-based Compulsive Sexual Behavior - Lack of Control, AVS_R_T Revision of the Asian Values- Traditional norms, AVS_R_F Revision of the Asian Values- Freedom & Flexibility, ATSPPH_SF_N Attitude Toward Seeking Psychology Profession Help Value and Need in Seeking Treatment, ATSPPH_SF_ O Attitudes Toward Seeking Professional Psychological Help - Openness to Seeking Help.

**Table 3** Heterotrait-Monotrait Ratios (HTMT) of correlations among the subfields of compulsive sexual behavior, Asian values, and attitudes toward seeking professional psychological help (N = 359).

| **Subscale** | ICSB_NA | ICSB_UC | ICSB_LC | AVS_R_T | AVS_R_F | ATSPPH_SF_N | ATSPPH_SF_O |
| --- | --- | --- | --- | --- | --- | --- | --- |
| ICSB_NA |  |  |  |  |  |  |  |
| ICSB_UC | 0.904  [0.846, 0.955] |  |  |  |  |  |  |
| ICSB_LC | 0.708  [0.620, 0.790] | 0.821  [0.762, 0.875] |  |  |  |  |  |
| AVS_R_T | 0.352  [0.256, 0.470] | 0.310  [0.253, 0.427] | 0.264  [0.226, 0.374] |  |  |  |  |
| AVS_R_F | 0.120  [0.124, 0.223] | 0.176  [0.163, 0.291] | 0.125  [0.138, 0.223] | 0.436  [0.405, 0.560] |  |  |  |
| ATSPPH_SF_N | 0.391  [0.278, 0.540] | 0.383  [0.284, 0.532] | 0.430  [0.319, 0.575] | 0.422  [0.361, 0.637] | 0.414  [0.366, 0.596] |  |  |
| ATSPPH_SF_O | 0.180  [0.115, 0.310] | 0.129  [0.105, 0.248] | 0.207  [0.150, 0.329] | 0.301  [0.236, 0.453] | 0.340  [0.283, 0.469] | 0.501  [0.406, 0.682] |  |

**Abbreviations:** ICSB_NA Individual-based Compulsive Sexual Behavior - Negative Affect, ICSB_UC Individual-based Compulsive Sexual Behavior- Unwanted consequences, ICSB_LC Individual-based Compulsive Sexual Behavior - Lack of Control, AVS_R_T Revision of the Asian Values- Traditional norms, AVS_R_F Revision of the Asian Values- Freedom & Flexibility, ATSPPH_SF_N Attitude Toward Seeking Psychology Profession Help Value and Need in Seeking Treatment, ATSPPH_SF_ O Attitudes Toward Seeking Professional Psychological Help - Openness to Seeking Help.

**Table 4** Collinearity Statistics.

|  | **VIF** |
| --- | --- |
| AVS_R_F -> ATSPPH_SF_O | 1.611 |
| AVS_R_T -> ATSPPH_SF_N | 1.197 |
| ICSB_LC -> ATSPPH_SF_O | 2.655 |
| ICSB_LC -> AVS_R_F | 2.686 |
| ICSB_NA -> ATSPPH_SF_N | 1.451 |
| ICSB_NA -> AVS_R_F | 2.913 |
| ICSB_NA -> AVS_R_T | 1.336 |
| ICSB_UC -> ATSPPH_SF_O | 3.296 |
| ICSB_UC -> AVS_R_F | 3.948 |

**Abbreviations:** ICSB_NA Individual-based Compulsive Sexual Behavior - Negative Affect, ICSB_UC Individual-based Compulsive Sexual Behavior- Unwanted consequences, ICSB_LC Individual-based Compulsive Sexual Behavior - Lack of Control, AVS_R_T Revision of the Asian Values- Traditional norms, AVS_R_F Revision of the Asian Values- Freedom & Flexibility, ATSPPH_SF_N Attitude Toward Seeking Psychology Profession Help Value and Need in Seeking Treatment, ATSPPH_SF_ O Attitudes Toward Seeking Professional Psychological Help - Openness to Seeking Help.

**Table 5** The mediating effect of Asian values and the moderate role of sexual frequency, having stable sexual partner, frequency of watching pornography (in a week), times of watching pornography (in a week) (N = 359)

| **Hyp.** | **Path** | **β** | **Confidence Intervals 95%** | **t** | **p** |
| --- | --- | --- | --- | --- | --- |
| **Direct effect** | | | | | |
| H1 | ICSB -> AVS | | | | |
| H1.1 | ICSB_LC -> AVS_R_F | -0.321 | [0.469, -0.114] | 3.543 | < 0.001 |
| H1.2 | ICSB_NA -> AVS_R_F | -0.257 | [-0.418, -0.056] | 2.810 | 0.005 |
| H1.3 | ICSB_NA -> AVS_R_T | 0.294 | [0.196, 0.425] | 4.999 | < 0.001 |
| H1.4 | ICSB_UC -> AVS_R_F | 0.674 | [0.361, 0.849] | 4.963 | < 0.001 |
| H2 | AVS -> ATSPPH | | | | |
| H2.1 | AVS_R_F -> ATSPPH_SF_O | -0.274 | [-0.401, -0.162] | 4.464 | < 0.001 |
| H2.2 | AVS_R_T -> ATSPPH_SF_N | -0.220 | [0.378, -0.091] | 2.987 | 0.003 |
| H3 | ICSB -> ATSPPH | | | | |
| H3.1 | ICSB_LC -> ATSPPH_SF_O | 0.375 | [0.178, 0.535] | 3.816 | < 0.001 |
| H3.2 | ICSB_NA -> ATSPPH_SF_N | -0.269 | [-0.392, -0.141] | 4.174 | < 0.001 |
| H3.3 | ICSB_NA -> ATSPPH_SF_O | 0.071 | [0.016, 0.134] | 2.311 | 0.021 |
| H3.4 | ICSB_UC -> ATSPPH_SF_O | -0.227 | [-0.413, -0.001] | 2.168 | 0.030 |
| H4 | Pornography_Frequency_7days x ICSB_NA -> ATSPPH_SF_N | -0.116 | [-0.214, 0.002] | 2.132 | 0.033 |
| H5 | Pornography_Time_7days x AVS_R_T -> ATSPPH_SF_N | 0.129 | [0.014, 0.230] | 2.317 | 0.021 |
| H6 | Sexual_Frequency x ICSB_NA -> AVS_R_T | -0.093 | [-0.171, 0.003] | 2.095 | 0.036 |
| H7 | Stable_Partner x ICSB_UC -> AVS_R_F | 0.103 | [-0.000, 0.203] | 1.979 | 0.048 |
| **Indirect effect** | | | | | |
| H8 | ICSB -> AVS -> ATSPPH | | | | |
| H8.1 | ICSB_NA -> AVS_R_T -> ATSPPH_SF_N | -0.065 | [-0.133, -0.026] | 2.370 | 0.018 |
| H8.2 | ICSB_LC -> AVS_R_F -> ATSPPH_SF_O | 0.088 | [0.029, 0.147] | 2.895 | 0.004 |
| H8.3 | ICSB_NA -> AVS_R_F -> ATSPPH_SF_O | 0.071 | [0.016, 0.134] | 2.311 | 0.021 |
| H8.4 | ICSB_UC -> AVS_R_F -> ATSPPH_SF_O | -0.185 | [-0.280, -0.084] | 3.530 | < 0.001 |

**Abbreviations:** ICSB_NA Individual-based Compulsive Sexual Behavior - Negative Affect, ICSB_UC Individual-based Compulsive Sexual Behavior- Unwanted consequences, ICSB_LC Individual-based Compulsive Sexual Behavior - Lack of Control, AVS_R_T Revision of the Asian Values- Traditional norms, AVS_R_F Revision of the Asian Values- Freedom & Flexibility, ATSPPH_SF_N Attitude Toward Seeking Psychology Profession Help Value and Need in Seeking Treatment, ATSPPH_SF_ O Attitudes Toward Seeking Professional Psychological Help - Openness to Seeking Help.
